# A Novel *CRYGD* Mutation (p.Trp43Arg) Causing Autosomal Dominant Congenital Cataract in a Chinese Family

**DOI:** 10.1002/humu.21386

**Published:** 2011-01

**Authors:** Binbin Wang, Changhong Yu, Yi-Bo Xi, Hong-Chen Cai, Jing Wang, Sirui Zhou, Shiyi Zhou, Yi Wu, Yong-Bin Yan, Xu Ma, Lixin Xie

**Affiliations:** 1 State Key Laboratory Cultivation Base, Shandong Provincial Key Laboratory of Ophthalmology, Shandong Eye Institute, Qingdao University Eye CollegeQingdao, China; 2 National Research Institute for Family PlanningBeijing, 100081, China; 3 State Key Laboratory of Biomembrane and Membrane Biotechnology, School of Life Sciences, Tsinghua UniversityBeijing 100084, China; 4 Institute of Biophysics, Lanzhou UniversityLanzhou 730000, China; 5 Graduate school, Peking Union Medical CollegeBeijing, China; 6 World Health Organization Collaborating Centre for Research in Human ReproductionBeijing, China

**Keywords:** *CRYGD*, autosomal dominant congenital cataract, ADCC), structure

## Abstract

To identify the genetic defect associated with autosomal dominant congenital nuclear cataract in a Chinese family, molecular genetic investigation via haplotype analysis and direct sequencing were performed Sequencing of the *CRYGD* gene revealed a c.127T>C transition, which resulted in a substitution of a highly conserved tryptophan with arginine at codon 43 (p.Trp43Arg). This mutation co-segregated with all affected individuals and was not observed in either unaffected family members or in 200 normal unrelated individuals. Biophysical studies indicated that the p.Trp43Arg mutation resulted in significant tertiary structural changes. The mutant protein was much less stable than the wild-type protein, and was more prone to aggregate when subjected to environmental stresses such as heat and UV irradiation. © 2010 Wiley-Liss, Inc.

## INTRODUCTION

Cataract, characterized by opacification of all or part of the eye's crystalline lens (Reddy, et al., 2004) and one of the most common treatable cause of visual loss in humans, can be generally categorized as early onset (congenital or juvenile) and age-related (Vijaya, et al., 1997). The estimated prevalence of congenital cataract is 2.2–2.49 cases per 10,000 live births (Rahi and Dezateux, 2000), and approximately 50% of congenital cataracts are inherited (Lampi, et al., 1997).

There are over eleven crystallin genes, encoding over 95% of the water-soluble structural proteins present in the crystalline lens and representing over 30% of its mass. The crystallin family can be divided into three distinct groups, including α-crystallins (CRYAA and CRYAB), β- and γ-crystallins (CRYBA1/A3/A4/B1/B2/B3 and CRYGA/B/C/D/S), which are included in a superfamily of microbial stress proteins and share a common two-domain structure, composed of four “Greek-key” motifs. The unique spatial arrangement and solubility of crystallins are essential to the optical transparency and high refractive index of the lens. Modification of crystallins can disrupt their normal structure in the lens and cause cataracts (van Rens, et al.., 1991).

γ-Crystallins comprise up to 40% of the soluble proteins expressed in the lens, and the gene cluster is located on chromosome 2q33-q35. Previous studies showed that mutations in the *CRYGD* (MIM# 123690) gene were responsible for coralliform, aceuliform, and fasciculiform phenotypes of cataracts (Graw, 2009). The systematic mutational analysis performed by King's group indicated that the integrity of the hydrophobic core and the domain interface are crucial for the folding and stability of γD-crystallin (Flaugh, et al.., 2005a; Flaugh, et al.., 2005b; Kosinski-Collins and King, 2003; Moreau and King, 2009). In addition, efficient quenching of the Trp fluorescence has a crucial role in protecting the protein against UV irradiation-induced damage (Chen, et al.., 2009; Chen, et al.., 2006; Chen, et al.., 2008). However, the inherited mutations identified thus far in human γD-crystallin are mainly charged surface residues; no Trp mutations have been characterized in either human beings or the mouse model. In this study, we report a Trp mutation (p.Trp43Arg) identified in a Chinese three-generation pedigree with autosomal dominant congenital cataract, for the first time.

## MATERIALS AND METHODS

### Clinical evaluation and examinations

**Figure 1:**
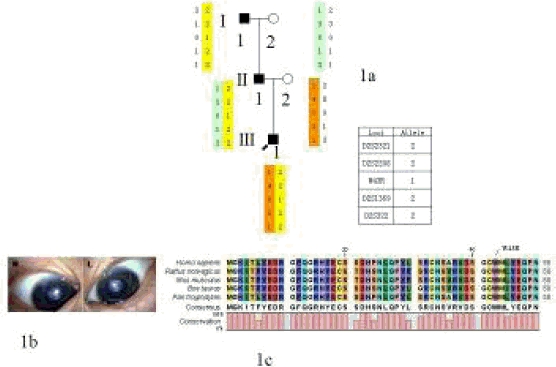
(a) The pedigree of a three-generation Chinese family with ADCC; Haplotype analysis of the family demonstrating segregation of four microsatellite markers and the mutation of 2q33-q35. (b) The proband, *III1*, presented with bilateral congenital nuclear cataracts, which consisted of a central nuclear opacity affecting the embryonic and fetal nucleus of the lens, while the cortex remained transparent. His corrected visual acuity was 0.4 for both eyes after surgery, and obvious nystagmus was also observed. (c) Conservation analysis of p.Trp43Arg of the *CRYGD* gene.

A three-generation Chinese family diagnosed with autosomal dominant congenital cataract (ADCC) was recruited at the Shandong Eye Institute (Qingdao, China). Three affected and two unaffected family members participated in the study (Figure 1a). All five family members underwent general physical examination and complete ophthalmic examinations, including refraction, corneal curvature, axial length, B scan ultrasonography, intraocular pressure, slit-lamp biomicroscopic, and fundus examination with dilated pupils, to identify whether there were any other ocular or systemic abnormalities.

Affected status was determined by a history of cataract extraction or ophthalmologic examination. The phenotypes were documented by slit lamp photography. A total of 200 unrelated control subjects with no family history of congenital cataracts were also recruited. Informed consent was obtained from all participants. The study protocol was in accord with the ethical guidelines of the 1975 Declaration of Helsinki and was approved by the Ethics Committees of the Shandong Eye Institute and the National Research Institute for Family Planning.

### DNA analysis and genotyping

Peripheral venous blood was collected for genomic DNA extraction using a QIAamp DNA kit (Qiagen, Valencia, CA) according to the manufacturer's instructions. PCR was performed with microsatellite markers close to candidate loci that were associated with congenital cataracts.

### Linkage analysis and haplotyping

Pedigree and haplotype data were analyzed using standard methods (Wang, et al.., 2009).

### Gene sequencing and bioinformatics

All coding exons and splice sites of sixteen known ADCC (nuclear phenotype) genes (*CRYAA, CRYAB, CRYBA1, CRYBB1, CRYBB2, CRYBB3, CRYGC, CRYGD, CRYGS, GJA3, GJA8, MIP, BFSP2, HSF4, FTL*, and *EYA1*) were amplified using specific primer pairs (primers for *CRYGD* are shown in Table 1, primers for the other genes are not shown). PCR products were sequenced using an ABI3730 Automated Sequencer (PE Biosystems, Foster City, CA).

**Table 1:** List of PCR primers

Candidate Gene-Primer ID	Primer sequence
CRYGD-1-F	5′- ATAGCAGGAGGGCTGCTG-3′
CRYGD-1-R	5′- ACATCCTCAAGTCAGGACC-3′
CRYGD-2-F	5′- AAGAAAGACACAAGCAAATCAGT-3′
CRYGD-2-R	5′- GCTTTTCTTCTCTTTTTATTTTCTGG -3′

The results were compared with sequences from the NCBI GenBank. Nucleotide numbering reflects the cDNA numbering, with +1 corresponding to the A of the ATG translation initiation codon in the reference sequence, according to journal guidelines (http://www.hgvs.org/mutnomen). The initiation codon is codon 1.

The possible functional impact of amino acid change was predicted using the PolyPhen (Polymorphism Phenotyping) program (http://genetics.bwh.harvard.edu/pph/).

### Protein expression, purification, and sample preparation

The full-length human CRYGD coding sequence was isolated from total cDNA of human lens cell by RT-PCR using Pfu polymerase and the following oligonucleotide primers: sense-primer (5′ TCAGAATTCATGGGGAAGATCACCCTCTA-3′), and antisense-primer (5′-TGACTCGAGTCAGGAGAAATCTATGACTCTCCT-3′). After digestion of the PCR product and of plasmid pET28a with NdeI and XhoI, the amplicon containing the coding sequence was ligated into the expression vector pET28a (Novagen). The resultant construct, pET-28a-CRYGD, was confirmed by DNA sequencing. Site-directed mutagenesis against Trp43 was carried out following standard procedures with the mutagenic primers listed below: 5′-GTGGACAGCGGCTGCCGGATGCTCTATGAGC-3′ and 5′-GCTCATAGAGCATCCGGCAGCCGCTGTCCAC-3′. The six-His Tag sequence of pET28a vector was fused to the N-terminus of the CRYGD open reading frame for further purification. The recombinant plasmids were transformed into E. coli BL21(DE3) Rossetta (Novagen). Overexpression and purification of the His-tagged proteins were performed as described previously (Gu, et al.., 2008; Pang, et al.., 2010). The final products were purified by affinity chromatography using Ni-NTA resin (Qiagen) and Hiload 16/60 Superdex 200 prep grade column on an AKTA purification system. Protein samples were prepared using 10 mM phosphate buffered saline (PBS) buffer, pH 7.0, with the addition of 1 mM DTT and 1 mM EDTA. The protein concentration was determined according to the Bradford method using bovine serum albumin as a standard (Bradford, 1976).

### Spectroscopic experiments

One-dimensional ^1^H-NMR experiments were performed on a Varian Unity Inova 500NB NMR spectrometer, and all data were processed and analyzed using the VNMR software provided by Varian Inc. The NMR samples were prepared by dissolving the protein in 10 mM phosphate buffered saline (PBS) buffer containing 1 mM DTT and 1 mM EDTA, pH 7.0, with the addition of 10% D_2_O. The NMR spectra were collected at 20°C using a spectral width of 8003.2 Hz (16 ppm) with 256 repetitions, a recycle delay of 1.5 s and a 45 degree pulse. The circular dichroism (CD), Trp intrinsic fluorescence, and ANS fluorescence were measured following previously described procedures (Pang, et al.., 2010). In brief, the fluorescence emission spectra were measured on a Hitachi F-2500 spectrofluorometer using 1-cm-pathlength cuvettes. The Trp intrinsic fluorescence was obtained using an excitation wavelength of 295 nm and an emission spectral range of 300-400 nm; while the ANS fluorescence experiments was performed with an excitation wavelength of 380 nm and an emission wavelength range of 400-600 nm. The final protein concentration was 0.2 mg/ml for most spectroscopic experiments, except for near-UV CD spectroscopy where 1 mg/ml was used.

### Size-exclusion chromatography (SEC) analysis

SEC analysis was performed using a Superdex G-200 column on an AKTA purification system. The column was equilibrated with at least two column volumes of 10 mM PBS buffer containing 1 mM DTT and 1 mM EDTA, pH 7.0. About 100 μl of protein solution at a concentration of 1 mg/ml was injected into the column. All samples were run at a flow rate of 0.4 ml/min at 4°C.

### Protein stability analysis

For the thermal stability, protein solutions with a final concentration of 0.2 mg/ml were incubated in a series of temperatures from 30°C to 86°C, and far-UV CD spectra and turbidity were measured for every 2°C followed with a two minute equilibration of the sample. Turbidity was monitored by measuring absorbance at 400 nm with an Ultraspec 4300 pro UV/Visible spectrophotometer using a 1 ml cuvette. The stability against UV irradiation was performed by exposing the protein solutions to UV light (30 W, 254 nm) on ice for 0-24 h. Buffer was used as a negative control. After being irradiated for a given period, the protein solutions were wrapped in tinfoil and kept under the same conditions until 24 h of incubation.

## RESULTS

### Clinical findings

The three-generation family, residing in an isolated region of north China, was identified with clear diagnosis of ADCC. Affected status was determined by a history of cataract extraction or ophthalmologic examination, and the affected individuals all presented with bilateral congenital nuclear cataracts, which consisted of a central nuclear opacity affecting the embryonic and fetal nucleus of the lens, while the cortex remained transparent (Figure 1b, the proband). The lens opacity was responsible for significant vision loss. All patients had undergone cataract extraction during childhood. The best corrected visual acuity ranged from 0.2 to 0.4 in the operated eyes. Marked nystagmus and amblyopia were observed in all the patients, even in the proband, who received phacoemulsification surgery for both eyes at 5 months old. They also had posterior scleral staphyloma. There was no history of other ocular or related systemic abnormalities in the family.

### Mutation and haplotype analysis

By sequencing sixteen known genes (all coding exons and splice sites) for ADCC (nuclear phenotype) and haplotype analysis, only one non-synonymous potential pathologic variant was identified in the exon 2 (c. 127T>C) of *CRYGD* (RefSeq NM_006891.3), which resulted in a substitution of Tryptophan to Arginine at codon 43 (p.Trp43Arg).

The alteration was not found in the 200 unrelated control subjects from the same Northern Chinese population (data not shown) or in the 1000 Genome Project dataset (http://browser.1000genomes.org/index.html). Meanwhile, haplotype analysis suggested that the region in which *CRYGD* is located in might be responsible for the disease (Figure 1a).

Polyphen predicted, with high confidence, that the p.Trp43Arg alteration in the CRYGD protein would be damaging. Furthermore, the mutation was located within a highly conserved region, as assessed by multiple-sequence alignment (Figure 1c).

These results demonstrated that, at the least, this variant could be a potential pathological mutation for this family.

### Effect of the p.Trp43Arg mutation on the secondary and tertiary structures of γD-crystallin

**Figure 2:**
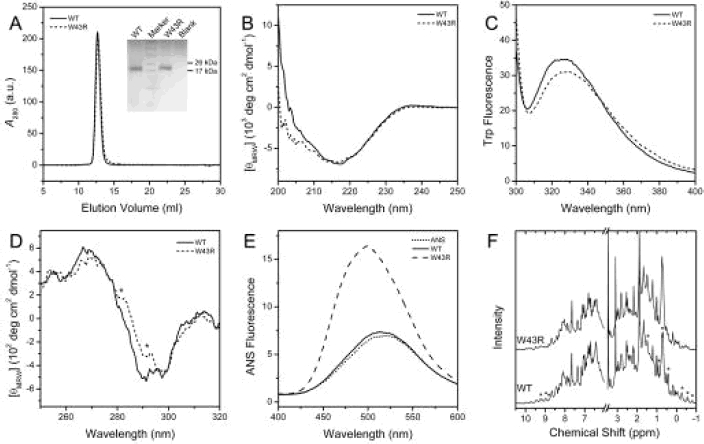
Effect of the p.Trp43Arg mutation on γD-crystallin structure by SEC (A) far-UV CD (B) intrinsic Trp fluorescence (C) near-UV CD (D) ANS fluorescence (E) and ^1^H-NMR (F). The proteins were dissolved in 10 mM PBS buffer containing 1 mM DTT and 1 mM EDTA, pH 7.0. The inset in panel (A) shows the SDS-PAGE analysis of the purified proteins. In panels (D) and (F), the significant changes in the CD and NMR signals are highlighted by asterisks.

To investigate how the p.Trp43Arg mutation could lead to autosomal dominant congenital cataract, the recombinant WT and mutated γD-crystallin were overexpressed in E. coli, purified, and studied by biophysical methods. Most of the overexpressed proteins were found in the soluble fraction when separated by centrifugation after lysis. The purified proteins were found to be homogenous and existed as a monomer in solution (Figure 2a). The almost identical elution volume on the SEC profile suggested that the p.Trp43 Arg mutation affects neither the oligomeric state nor the SEC radius of γD-crystallin. The single negative peak at ∼217 nm in the far-UV CD spectra (Figure 2b) of both proteins indicated that both proteins were mainly composed of β-sheet structures, which is consistent with the crystal structure of γD-crystallin (Basak, et al.., 2003). The similarity in the curve shape and mean residue ellipticity suggested that the mutation did not affect the percentages of secondary structure contents of γD-crystallin.

Intrinsic Trp fluorescence, extrinsic ANS fluorescence, and NMR experiments were carried out to examine the effects of the p.Trp43Arg mutation on γD-crystallin tertiary structure. There are four Trp residues in γD-crystallin, and the intrinsic Trp fluorescence is predominantly contributed by Trp43 and Trp131 fluorophores; the maximum emission wavelength (E_max_) of the WT protein is at 326 nm (Flaugh, et al.., 2005a). A previous study indicated that the E_max_ of Trp43 and Trp131 were at 327 run and 318 nm, respectively (Kosinski-Collins and King, 2003). The results in Figure 2c indicated that the p.Trp43Arg mutation had led to a red shift of the E_max_ to ∼ 329 nm, implying that the mutation might result in an alternation in the microenvironments of the other three Trp residues. This deduction was further verified by the near-UV CD spectroscopy, which is a sensitive tool for monitoring the microenvironments of aromatic residues (Kelly, et al.., 2005). As indicated by the asterisks in Figure 2d, there is more than one Trp residue whose microenvironments were affected by the mutation. Meanwhile, changes in the tertiary structure of γD-crystallin were also reflected by a gradual increase in the ANS fluorescence, which indicated hydrophobic exposure by specifically binding of the ANS molecules to the protein (Cardamone and Puri, 1992). 1H-NMR spectroscopy was used to investigate the effect of the p.Trp43Arg mutation on γD-crystallin structure at the atomic level. As shown in Figure 2f, the mutation slightly decreased the dispersion of the NMR peaks, indicating that the structure of the mutant was not as compact as the WT protein. More importantly, significant differences were observed for NMR signals from both the -CH_3_ groups of the amino acid side chains (from -1 ppm to 1 ppm) and -NH groups (>7.5 ppm) at the backbone of the protein, implying that the overall structure of γD-crystallin was modified by the mutation. Thus, the spectroscopic experiments indicated that the p.Trp43Arg mutation impaired the wild-type structure of γD-crystallin in solution.

### Effect of the p.Trp43Arg mutation on the structural stability of γD-crystallin

**Figure 3:**
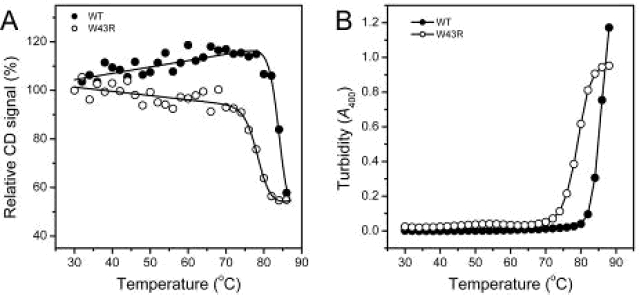
Thermal stability of the WT and p.Trp43Arg γD-crystallin evaluated by far-UV CD (A) and turbidity experiments (B). The CD data were normalized by taking the ellipticity of the proteins measured at 30°C as 100%.

To gain insight into the impact of the p.Trp43Arg mutation on the stability and aggregation of γD-crystallin, we performed thermal unfolding analysis monitored by far-UV CD and turbidity. As shown in Figure 3, the CD signal of the WT protein increased slightly with rising temperature from 30°C to 78°C, and the main transition was observed between 78°C and 86°C, with a midpoint temperature of 84.3±0.8°C when fitted to a two-state model. The mutant had a significantly lower thermal stability, and deviations could clearly be observed between the transition curves of the two proteins. The ellipticity of the mutant decreased with increasing temperature, and the midpoint temperature of the main transition was 77.8±0.7°C. The turbidity experiments indicated that the WT protein began to aggregate at 82°C, while the mutant began to aggregate at 72°C.

**Figure 4:**
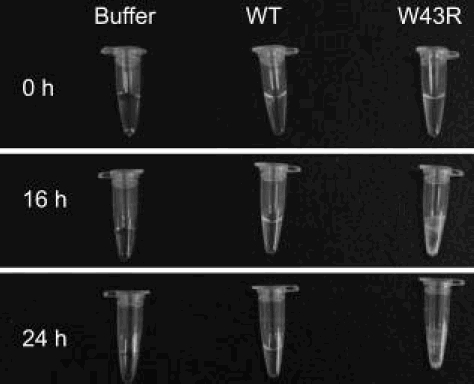
Stability of the WT and p.Trp43Arg γD-crystallin against UV irradiation. The protein samples were exposed to UV light for a given period, and then the treated samples were wrapped in tinfoil and kept under the same conditions until 24 h of incubation. A tube containing buffer in the absence of proteins was used as the negative control.

Both the WT and mutated proteins, at a final concentration of 5 mg/ml, were rather stable when incubated at 37°C, and no aggregates were observed for at least two weeks (data not shown). To further investigate whether the changes in the structure and thermal stability affected γD-crystallin's solubility under physiologically relevant conditions, long-term UV irradiation was applied to the protein solutions. Both WT and mutated protein solutions were as clear as the buffer without UV irradiation. After 16 h of treatment, the WT protein remained soluble, whereas the mutant showed amorphous aggregates that could be observed by eye. After 24 h of irradiation, no significant difference was observed between the WT γD-crystallin and the buffer, whereas serious deposition occurred in the p.Trp43Arg γD-crystallin. Thus, the results presented in Figures 3 and 4 indicate that the mutation decreased protein stability and promoted protein aggregation of γD-crystallin when subjected to stress.

## DISCUSSION

Nuclear cataract refers to opacification within the embryonic and/or fetal nuclei of the lens. The lens opacities vary from small pulverulent (“dustlike”) opacities, punctate dots, and blue dots to completely confluent nuclear opacification. Prominent opacification of the anterior and posterior Y sutures may occur. To date, eight genes for nuclear cataract have been identified (*CRYBA1, CRYAA*, and *CRYGD*, which are related with the nuclear phenotype but not the pulverulent, and *CX46, CX50, CRYBB2, CRYBB3* and *CRYGC*, which are candidates for nuclear pulverulent only). In the lenses of individuals of less than two years of age, the fraction of γ-crystallins was reported to be 35% *CRYGS*, 45% *CRYGC*, and 20% *CRYGD* (Siezen, et al.., 1987).

Other mutations of *CRYGD* have been reported. Functional analysis of a p.Arg14Cys (R14C) variant (Stephan, et al.., 1999) revealed that the R14C forms disulfide-linked oligomers, which markedly raised the phase separation temperature of the protein solution so that R14C precipitated gradually. Plotnikova et al.. (Plotnikova, et al.., 2007) discovered a c.70C>T transition that resulted in a p.Pro23Ser (P23S) substitution and a p.Pro23Thr replacement was found in a congenital lamellar cataract (Nandrot, et al.., 2003). Kmoch, et al.. noted a c.109C>A (p.Arg36Ser) mutation (Kmoch, et al.., 2000), and a G-to-A transition at nucleotide 176 was found by Heon et al.. (p.Arg58His) (Heon, et al.., 1999). Functional assays of p.Arg36Ser and p.Arg58His indicated that these mutations do not alter the folding of the protein but alter the surface characteristics of *CRYGD*. They lower its solubility and enhance the crystal nucleation rate and their precipitation. In at least one case, crystals were formed in the lens (Pande, et al.., 2001). A G-to-A transition at nucleotide 470 (p.Trp156X) was also reported (Santhiya, et al.., 2002).

The mutation identified in this study, p.Trp43Arg, is possibly a novel pathogenic non-synonymous mutation causing the ADCC nuclear phenotype. The p.Trp43Arg substitution is likely to cause cataracts because it segregated with the phenotype and was not detected in either the unaffected family members or in the 200 ethnically matched controls. Trp43 is located in the highly conserved region of γD-crystallin (Figure 1d), and is also located in the hydrophobic core of the N-terminal domain (Basak, et al.., 2003). The p.Trp43Arg mutation resulted in significant tertiary structural changes, as evidenced by NMR, near-UV CD, intrinsic Trp fluorescence, and extrinsic ANS fluorescence, although no difference was observed in the secondary structure components of γD-crystallin (Figure 2). It was possible that the substitution of Trp43 by a charged residue Arg disrupted the integrity of the hydrophobic core of the conserved crystallin domain composed of four Greek keys, which is believed to be crucial to γD-crystallin folding and stability (Moreau and King, 2009). The p.Trp43 Arg mutant was less stable than the WT protein, and was much more prone to aggregation when subjected to environmental stresses (Figures 3 and 4). In particular, the WT protein was considerably resistant to UV irradiation, while the p.Trp43 Arg mutant was easily destabilized and tended to form large aggregates (Figure 4). This observation was in agreement with the proposal that the Trp residues in γD-crystallin protect the eyes against UV damage by absorbing the UV light and quenching the fluorescence (Chen, et al.., 2009; Chen, et al.., 2006; Chen, et al.., 2008).

In summary, we described a human nuclear congenital cataract caused by a novel mutation, which substituted an amino acid at position 43 in the CRYGD gene. The linkage between the mutation and the onset of cataract was also characterized by the prominent effects of the p.Trp43Arg mutation on γD-crystallin structure and stability. To our knowledge, the current study is the first to suggest that the CRYGD gene is involved in the etiology of Chinese ADCC. Evidence from the previous and present studies leads us to believe that CRYGD plays an important role in the pathogenesis of ADCC in humans.
